# French national survey of dog and cat owners on the deworming behaviour and lifestyle of pets associated with the risk of endoparasites

**DOI:** 10.1186/s13071-019-3712-4

**Published:** 2019-10-14

**Authors:** Clarisse Roussel, Jason Drake, Juan Manuel Ariza

**Affiliations:** 1 Elanco Animal Health, Lilly House, 24 boulevard Vital Bouhot, CS 50004, 92521 Neuilly sur Seine Cedex, France; 2 Elanco Animal Health, 2500 Innovation Way, Greenfield, IN 46140 USA; 3 Independent Researcher, Nantes, France

**Keywords:** ESCCAP, Deworming recommendations, Risk factors, Helminths, Zoonosis, Companion animals, France

## Abstract

**Background:**

Endoparasites in dogs and cats are a concern related to pet health and zoonotic risks. Several determinants may affect the endoparasite transmission and infection of dogs and cats such as pet’s lifestyle or regional parasite distribution. Although different zoonotic endoparasites, such as *Toxocara* spp. and *Echinococcus* spp., have been identified in France, little information exists about the deworming behaviors of owners or the frequency of occurrence of risk factors associated with endoparasite infection or transmission. Deworming guidelines, such as those created by the European Scientific Counsel Companion Animal Parasites (ESCCAP), recommend a deworming frequency according to the risk of infection of every pet and the potential risk for zoonotic transmission. The objectives of this study were to explore how lifestyles of dogs and cats from France were related to a particular risk of endoparasites and assess whether deworming frequencies complied with ESCCAP recommendations.

**Methods:**

French data were extracted from a database created during a recent European pet owner survey regarding endoparasitic infection risk. Dogs and cats were grouped into risk categories based upon the ESCCAP guidelines. The compliance between the actual and recommended deworming frequencies were explored among the regions surveyed.

**Results:**

The majority of dogs and cats were older than 6 months, had outdoor access, had contact with children or elderly people, and lived in rural and town areas. Most of the dogs were in contact with other dogs, snails or prey (83%), and ate slugs, snails, grass or dug in the garden (68%). Likewise, most of the cats hunted outside (57%) and caught prey animals (52%). Consequently, most of the dogs (89%) and cats (53%) were considered to be in the highest-risk category (D). However, independent of the region, the average deworming compliance for dogs was poor (6%). While deworming compliance for cats in category A (low-risk) was excellent (94%), for cats in category D it was poor (6%).

**Conclusions:**

Deworming compliance is needed to enhance pet health and reduce zoonotic risks. Future studies are warranted to thoroughly investigate the compliance and effectiveness of deworming protocols, and the risk factors associated with endoparasites in France.

## Background

Endoparasites, particularly helminths, infect an important number of dogs and cats in Europe. Depending on the parasite involved, the severity of the clinical conditions associated with infection may vary from slight gastrointestinal signs to life-threatening situations [1, 2]. Moreover, some parasites represent a significant concern for public health because of their zoonotic potential. Recent European reports have revealed endoparasite prevalences ranging between 9–69% in dogs [3–14] and 8–40 % in cats [3, 5–8, 13, 15, 16]. However, the prevalence of endoparasites is a specific population measure at a given time that is affected by several determinants including the design of the study [17], the season [18, 19], the geographical location [12, 20], the tools implemented for the diagnosis [21], and the lifestyle of the population studied (household or shelter animals) [9–11, 14]. In France, scarce data are available about endoparasite prevalence in dogs and cats, especially for helminths, with the last national report from 1997 reporting that 22% of dogs and 17% of cats were infected by endoparasites. Moreover, there is little information about the main factors influencing endoparasites in small animals in France [20].

Among zoonotic helminths present in France, *Toxocara* spp. and *Echinococcus* spp. are some of the most important infecting dogs and cats. In environments highly contaminated by both parasites, humans typically become infected by ingestion of infective eggs. Responsible for human toxocariasis, *Toxocara* spp. is the most frequent nematode found in dogs (9.7%) and cats (14.3%). Whereas infection rates of *Echinococcus multilocularis* in dogs (0.4%) and cats (1.5%) appear to be low, prevalence of more than 10% has been reported in red foxes (main final host) in France, being one of the highest in Europe [22]. During the last decade, the parasite has spread over 25 new localities within France, including southern regions and urban areas like Paris [23, 24]. *Echinococcus multilocularis* is responsible for the dangerous alveolar echinococcosis (AE) in humans. Although, historically is considered a rare disease, the number of cases has increased in Europe, particularly in France, during the past few decades [25–27]. Concerning *Echinococcus granulosus*, responsible for the cystic echinococcosis (CE) in humans, little is known about its prevalence in French dogs. Nevertheless, data from intermediate hosts (sheep, cattle, pigs, goats, horses or cervids) collected in slaughterhouses confirming that the parasite is present across the country, though in a low prevalence [28–30].

While *Toxocara* spp. and *Echinococcus* spp. represent major zoonotic threats to public health, they have a smaller impact on the animal’s health (none to mild signs of disease). Conversely, helminths such as *Angiostrongylus vasorum* and *Dirofilaria immitis*, represent an important threat for the welfare and health of small animals in France. Although *A. vasorum* and *Dirofilaria* spp. were previously considered of low prevalence in Europe, different factors have allowed the spread of those parasites, including: (i) climate change and the consequent ecological changes on intermediate hosts (i.e. slugs, snails for *A. vasorum* and mosquitoes for *D. immitis*); (ii) increased trade and movement of pets; and (iii) the reservoir-vector diversification (i.e. *Aedes albopictus*) [2, 19]. *Angiostrongylus vasorum* is primarily a parasite of Canidae, being mainly responsible for cardiorespiratory signs and less frequently for coagulopathies and neurologic disorders [2]. The parasite prevalence in France ranges from 1.4% to 11.8% [18, 31, 32]. *Dirofilaria immitis* affects mainly dogs, however other hosts, such as cats, ferrets, foxes and wolves, may be affected as they can be involved in the live-cycle of the parasite [1, 19]. Dogs infected with the parasite develop a progressive cardiopulmonary disease [1]. In France, the parasite is distributed in the southern regions, the Corse and in some French overseas territories (Martinique and French Guyana among others) with reporting prevalence rates ranging between 0.22–6.8% [19, 33, 34]. Regarding its zoonotic potential, the parasite is responsible for the benign pulmonary dirofilariasis in humans. *Dirofilaria repens*, in contrast, is responsible for ocular and cutaneous dirofilariasis in humans, with a number of cases that have increased recently (23 cases reported between 1995 and 1999 versus 63 cases between 2000 and 2011 in France) [1, 35–37]. Small animals infected typically present minor or even no signs of disease [1, 19, 38]. Additionally, it is worth mentioning that in France, the parasite *Thelazia callipaeda* have been recently identified throughout the south-western region of the country [39]. The distribution of this parasite is related to the presence of the intermediate host, the fruit fly *Phortica variegata*. This eyeworm can cause ocular problems such as blepharospasm, epiphora, conjunctivitis, keratitis and corneal ulcers in dogs, cats, foxes and lagomorphs. Although *T. callipaeda* is a recognized agent of a zoonosis responsible for similar ocular symptoms as seen in animals, only a few human cases have been reported in France [40].

In order to control the zoonotic risk posed by endoparasites, and to increase the welfare and the health of small companion animals, ESCCAP has developed deworming guidelines aimed to reduce the environmental infection pressure and parasitic infections of small animals. These guidelines are based on the main risk factors associated with endoparasites identified across the scientific evidence on endoparasite control of dogs and cats. According to ESCCAP, the assessment of the lifestyle and the physiological status of animals should guide veterinarians in the deworming decision-making process. Currently, the ESCCAP guidelines propose four main categories (A, B, C and D) for dogs and two main categories (A and B) for cats associated with particular risk factors and advise a deworming frequency for each category [41]. Nevertheless, even with available guidelines, owners and veterinarians do not always follow the experts’ recommendations [42], suggesting that there is a lack of concern or awareness regarding the parasites and the risks associated with infection.

To explore the endoparasitic risks associated with the lifestyle of dogs and cats in France, we explored and analysed the results of a recent European survey on small companion animals lifestyle and pet owners deworming behavior. Additionally, we explored whether pet owners’ deworming behavior in France follows the recommended deworming frequency.

## Methods

### Study design

The lifestyle of dogs and cats and the deworming behavior of pet owners were explored through an online survey conducted in five European countries in July 2017 [43]. The information recorded from the French territories is presented and investigated at regional levels in this manuscript.

The methodology implemented in the survey has been described in detail in the previous publication [43]. Briefly, the surveyed cat or dog owners were at least 18 years-old, were responsible for the pet health care of fewer than 10 animals and owned pets examined by a veterinarian at least once a year. Owners who used their animals for any professional reasons were excluded from the survey. These criteria were established to homogenize the studied population to typical pet owner households. In cases where an owner owned both a cat and dog, the owner was randomly provided the survey of only one species.

A total of 19,855 French owners were recruited from a database panel through a link to fulfill the online survey in order to achieve a representative sample composed by 1000 owners of 500 cats and 500 dogs according to recent demographic statistics about pet-owning households. Small incentives were offered to owners to encourage survey completion. Once the quotas of 500 cat owner and 500 dog owner surveys were reached, the online survey was closed to prevent further submissions.

The survey questionnaire (Table 1) was created to allow classification of dogs and cats into 4 risk categories according to their lifestyle, additional factors affecting their exposure and infection, and potential zoonotic concerns. ESCCAP suggests 4 risk groups for dogs and only 2 for cats. To facilitate direct comparison between cats and dogs as well as group higher risk cats into the appropriate deworming frequency, the ESCCAP risk groups for cats (A-B) were converted into four risk groups (A-D) using the additional risk factors outlined in the ESCCAP guidelines [41]. For each category, an ESCCAP recommended deworming frequency is associated (Table 2). The questionnaire comprised general questions, including one related to the characteristics and the geo-localization of the responder’s residence, one about deworming frequency, and six (cats) or eight (dogs) related to the lifestyle of their cat or dog. Additional questions related to commercial deworming products and the relationship between owners and veterinarians were included in the survey but were not explored in this paper. The questions related to the lifestyle of the animals and the deworming behavior of owners were dichotomous (answers Yes or No). Questions related to owner behavior were placed at the beginning of the questionnaire in order to avoid potential influence on the rest of the questions. Surveys were confidential and each owner was informed of the purpose of the survey and accepted the terms of the study.

**Table 1:** Questionnaire for dog and cat owners

Question number	Question for dog owners	Question for cat owners
1	Which of the following categories describes best the place you live in? Rural area, town, suburban, city?	Which of the following categories describes best the place you live in? Rural area, town, suburban, city?
2	How often do you currently deworm your dog?	How often do you currently deworm your cat?
3	Is your dog less than 6 months-old?	Is your cat less than 6 months-old?
4	Is your dog exercising only in your own garden? (no contact with public places, other dogs, slugs, snails, raw meat or prey animals)	Is your cat kept indoors all the time (and does not eat raw meat)?
5	Does your dog exercise off the lead?	Does your cat hunt outside?
6	Does your dog ever get in contact with other dogs, slugs, snails or prey animals?	Does your cat ever catch prey such as mice and birds?
7	Does your dog eat slugs, snails, grass or dig in the garden?	Does your cat eat raw meat?
8	Does your dog ever catch animals such as rabbits or mice or pick up carcasses?	Does your cat interact with children or the elderly?
9	Does your dog eat any raw meat?	
10	Does your dog interact with children or the elderly?

**Table 2:** Dog and cat risk category definitions

Risk category	Dogs		Cats	
	Description	EU ESCCAP recommended deworming frequency^b^	Description	EU ESCCAP recommended deworming frequency^b^
A	Older than 6 months, lives indoors only or goes outdoors but has no direct contact with parks, sandpits, playgrounds, (faeces from) other dogs, snails and slugs, raw meat or prey	1–2 times per year	Cat lives indoors. Infection pressure with worm stages is low, eating rodents unlikely	1–2 times per year
B	Older than 6 months, goes outdoors and has direct contact with parks, sandpits, playgrounds, and (faeces from) other dogs and cats; but does not eat prey animals and/or snails and slugs and/or goes outdoors to hunt and does not eat raw meat	4 times per year	Cat goes outdoor. Infection pressure with worm stages is high, eating rodents likely	4 times a year
C^a^	Older than 6 months, goes outdoors and has direct contact with parks, sandpits, playgrounds, and (faeces from) other dogs and cats and eats prey animals and/or snails and slugs and/or goes outdoors to hunt and eats raw meat	> 4 times per year	Cat eats prey animals and/or goes outdoors to hunt and eats raw meat	more than 4 times per year
D^a^	Are less than 6 month-old, or eats prey animals and/or goes outdoors to hunt, or lives indoors, eats raw meat and lives with children/elderly	12 times per year	Are less than 6 months old, or cat is free to roam outdoors and shares home with young children or immunocompromised individuals	12 times per year

^a^ESCCAP Cat Risk Categories include A and B only. Additional risk factors in the ESCCAP guidelines were used to create Groups C and D for consistency in reporting and comparison of dog and cat results

^b^Or carry out faecal examination

### Data analysis

For each region, dogs and cats were grouped according to their lifestyles into one of the four risk categories created from the ESCCAP guidelines without taking into account the endemic risk associated with the presence of some parasites on the French territories (Table 2). The recommended deworming frequencies for each risk category were adapted based on local risk assessments (Table 3).

**Table 3:** Dog and cat recommended deworming frequencies in France

Dog/ Cat^a^	Dog	Cat
Puppy/ Kitten	Monthly	Monthly
Only exercised in garden, supervised/Indoor	1–2× yearly	1–2× yearly
na^b^/Outdoor	na^b^	4× yearly
Catches animals/ Eats prey	Monthly	> 4× yearly
With children, elderly	Monthly	Monthly
Allowed off-lead/ na^b^	> 4× yearly	na^b^
Fed, eats raw meat	> 4× yearly	> 4× yearly
Eats slugs, snails/ na^b^	Monthly	na^b^

^a^if “Yes” in survey

^b^na: not applicable

To determine the compliance between the current owner deworming practices and the ESCCAP deworming recommendations, the proportions of dogs and cats dewormed complying with the deworming recommendations were calculated for each region. In general, animals were considered compliantly dewormed if: (i) animals in category A were dewormed at least once a year; (ii) animals in category B were dewormed at least 3 times per year; (iii) animals in category C were dewormed at least 5 times per year; and finally, (iv) animals in category D were dewormed at least 6 times a year. According to the proportion of dogs and cats following the deworming recommendations, in each region compliance was considered as excellent (> 90%), good (60–90%), moderate (40–60%) and poor (< 40%).

### Translation

French translation of the Abstract is provided in Additional file 1.

## Results

From the 19,855 French owners contacted, only 1984 followed the invitation and visited the entry page. Of those, 71 cancelled the survey before completion. Additionally, 738 surveys did not match the inclusion criteria. Finally, 175 were excluded after the database reached the targeted fixed population of 500 dog owners and 500 cat owners. On average, 38 dog owners and 38 cat owners responded the survey by region (responders ranged from 3 to 96). The Corse region was underrepresented with only three dog and three cat responders.

### Dogs

Table 4 presents the main lifestyles of dogs and the deworming behaviour of owners for each region. In general, independent of the region surveyed, most dogs: (i) were older than 6 months (97%); (ii) were kept on lead when outside (83%); (iii) were in contact with other dogs, snails or prey (83%); (iv) ate slugs, snails, grass or dug in the garden (68%); and (v) were in contact with children and/or elderly people (75%). Furthermore, most of the responders lived in rural areas (44%) or in towns (29%). The proportion of dogs having outdoor access in addition to their own garden varied between the regions from 43% (Nouvelle-Aquitaine) to 80% (Bretagne). Additionally, of the dogs having outdoor access, between 20% (Ile-de-France) and 45% (Bretagne) went “off lead”. Finally, the proportion of owners reporting that their dogs caught prey animals varied from 10% (Grand Est) to 30% (Hauts-de-France).

**Table 4:** Regional distribution and main characteristics of the dogs according to the survey responses collected from 500 French owners

Sample characteristic	Auvergne- Rhône-Alpes (*n *= 57)	Bourgogne- Franche- Comte (*n *= 27)	Bretagne (*n *= 30)	Centre- Val-de- Loire (*n *= 28)	Corse (*n *= 3)	Grand- Est (*n *= 39)	Hauts-de France (*n *= 56)	Ile-de-France (*n *= 57)	Normandie (*n *= 31)	Nouvelle- Aquitaine (*n *= 48)	Occitanie (*n *= 51)	Pays de la Loire (*n *= 35)	Provence-Alpes-Côte d’Azur (*n *= 38)	Total (*n *= 500)
Puppies (< 6 months-old)	3	2	3	0	0	1	4	1	0	1	2	0	0	17 (3%)
Garden only access	23	9	6	12	0	15	28	16	14	27	18	14	14	196 (39%)
Goes outside “off lead”	10	5	11	5	1	6	6	8	4	9	11	6	7	89 (17%)
Contact with other dogs, snails or preys	50	22	22	22	3	31	40	51	24	42	45	32	31	415 (83%)
Eat slugs, snails, grass or digs in garden	40	21	21	20	3	22	33	41	20	34	37	23	24	339 (68%)
Catch prey animals	14	4	8	7	3	4	14	13	7	12	11	8	7	112 (22%)
Feed raw meat and don’t hunt	8	6	0	1	1	4	2	4	6	5	6	5	4	52 (10%)
Contact with children and elderly	43	23	23	21	3	23	41	47	21	35	37	27	31	375 (75%)
Living area														
Rural area	28	17	17	15	2	18	25	6	14	26	28	15	11	222 (44%)
Town	12	7	12	6	1	13	19	10	8	11	17	11	17	144 (29%)
Suburban	9	1	1	5	–	3	7	25	5	10	2	4	4	76 (15%)
City	8	2	–	2	–	5	5	16	4	1	4	5	6	58 (12%)
Average dewormings per year in puppies	3	3	1.66	–	–	–	2.75	3	–	2	2.50	–	–	1.95
Average dewormings per year	2	3	2.46	2.25	2	2.69	1.91	2.19	2.58	2.60	2.33	2.42	1.50	2.28

**Fig. 1:**
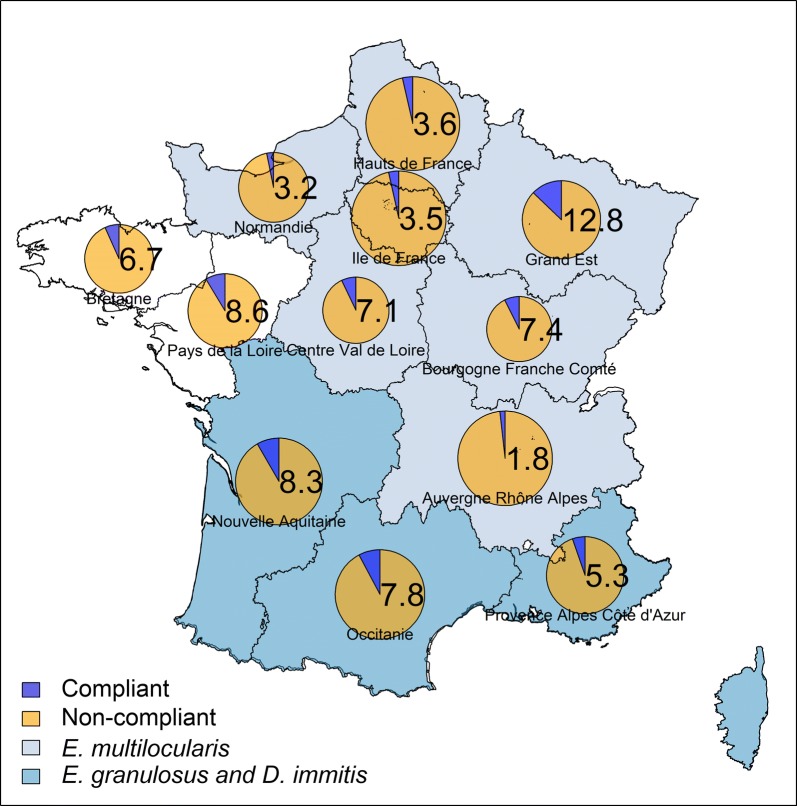
Proportion (%) of dog owners deworming in compliance with ESCCAP risk-based recommendations. Size of the pie is proportional to the size of sample surveyed. Regions are coloured according to the presence of parasites of zoonotic concern

Among the regions studied, dogs were dewormed between 1.50 and 3 times per year (2.28 on average) (Table 4). According to the risk category classification, 2%, 2%, 7% and 89% of the dogs were grouped into categories A (lowest risk), B, C and D (highest risk), respectively (Table 6). The overall compliance of dog owners with the deworming recommendations for each region is presented in Fig 1. It ranges from approximately 2% (Auvergne-Rhônes-Alpes) to 13% (Grand Est). For Category D, where most of the dogs were grouped, on average the compliance was poor (4%) within the regions fluctuating from 0% (Auvergne-Rhône-Alpes, Bretagne, Provence-Alpes-Côte d’Azur and Corse) to 13% (Grand Est). For the remaining categories (A, B and C) represented by small numbers of individuals, the overall compliance varied between poor for category C (0%) and category B (36%), to excellent for category A (100%). Finally, across France the average compliance with the recommended deworming was poor (6%).

### Cats

**Fig. 2:**
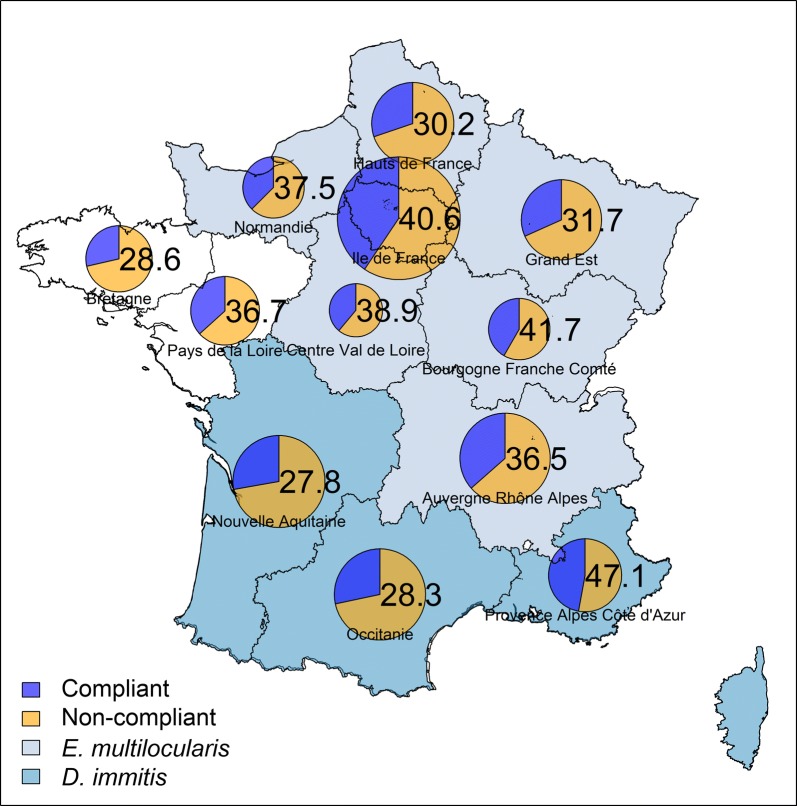
Proportion (%) of cat owners deworming in compliance with ESCCAP risk-based recommendations. Size of the pie is proportional to the size of sample surveyed. Regions are coloured according to the presence of parasites of zoonotic concern

Table 5 presents the main lifestyles of cats and the deworming behaviours of owners for each region. Most of the cats from the survey were older than 6 months (97%) and had outdoor access (66%). The cat owners mostly reported living in rural areas (31%) and towns (32%). While only 5% of the responders fed their cats raw meat, overall, 57% (33% to 68%) reported living with children or elderly people, 57% (33% to 72%) hunted outside and 52% (33% to 65%) caught prey animals. Among the regions studied, cats were dewormed between 1.33 and 3 times per year (2.25 on average). Across the country 33%, 3%, 11% and 53% cats were grouped in categories A (lowest risk), B, C and D (highest risk), respectively (Table 6). The overall compliance of cat owners with the deworming recommendations for each region is presented in Fig 2. Deworming compliance ranged from 28% (Nouvelle-Aquitaine, Occitanie) to 47% (Provence-Alpes-Côte d’Azur). Approximately half of the cats from the survey were in category D for which compliance was poor (6%) across all regions fluctuating from 0% (Centre-Val-de-Loire, Occitanie, Pays de la Loire and Corse) to 13% (Bretagne and Normandie). For the cats grouped in category A the compliance was excellent (94%). The remaining classes were composed by smaller numbers of individuals and the overall compliance was poor (category C 7.5% and category B 20%). In conclusion, across France the average compliance with deworming recommendations was poor (36%).

**Table 5:** Regional distribution and main characteristics of the cats according to the survey responses collected from 500 French owners

Sample characteristic	Auvergne- Rhône-Alpes (*n *= 52)	Bourgogne- Franche- Comte (*n *= 24)	Bretagne (*n *= 28)	Centre- Val-de- Loire (*n *= 18)	Corse (*n *= 3)	Grand- Est (*n *= 41)	Hauts-de France (*n *= 43)	Ile-de-France (*n *= 96)	Normandie (*n *= 24)	Nouvelle- Aquitaine (*n *= 54)	Occitanie (*n *= 53)	Pays de la Loire (*n *= 30)	Provence-Alpes-Côte d’Azur (*n *= 34)	Total (*n *= 500)
Kittens (< 6 months-old)	2	0	0	1	0	3	0	3	0	3	2	0	1	15 (3%)
Keep indoors all the time	18	9	5	8	2	12	14	41	8	13	14	11	16	171 (34%)
Hunt outside	31	13	19	10	1	23	24	48	13	39	34	15	17	287 (57%)
Catch prey animals	30	11	18	8	1	23	21	41	13	35	31	15	15	262 (52%)
Feed raw meat	4	1	0	0	0	4	4	4	0	2	2	2	0	23 (5%)
Contact with children and elderly	32	14	12	12	1	18	29	50	15	31	36	18	16	284 (57%)
Living area														
Rural area	17	9	8	6	2	17	16	9	10	24	19	11	6	154 (31%)
Town	16	8	15	5	–	11	16	20	9	19	18	9	13	159 (32%)
Suburban	7	1	2	3	1	6	8	38	1	8	7	8	5	95 (19%)
City	12	6	3	4	–	7	3	29	4	3	9	2	10	92 (18%)
Average dewormings per year in kittens	1.50	–	–	2	–	5.66	–	8.66	–	3.66	1.50	–	2	4.26
Average dewormings per year	2.65	2.08	2	1.66	1.33	2.58	1.90	2.20	3	2.40	2.05	2.20	2.11	2.25

**Table 6:** National and regional distribution of dogs and cats according to the risk category classification implemented

Region	Dog					Cat				
	Risk category, *n* (%)					Risk category, *n* (%)				
	A	B	C	D	*N* (total)	A	B	C	D	*N* (total)
Auvergne-Rhône-Alpes	1 (2)	2 (3)	10 (18)	44 (77)	57	15 (29)	3 (6)	10 (19)	24 (46)	52
Bourgogne-Franche-Comte	0	0	0	27 (100)	27	9 (37)	0	0	15 (63)	24
Bretagne	1 (3)	1 (3)	4 (13)	24 (80)	30	7 (25)	3 (11)	10 (36)	8 (29)	28
Centre-Val-de-Loire	0	3 (11)	4 (14)	21 (75)	28	7 (39)	2 (11)	3 (17)	6 (33)	18
Corse	0	0	0	3 (100)	3	2 (67)	0	0	1 (33)	3
Grand-Est	2 (5)	0	0	37 (95)	39	12 (29)	0	28 (68)	13 (30)	41
Hauts-de France	1 (2)	0	0	55 (98)	56	13 (30)	0	3 (7)	27 (63)	43
Ile-de-France	0	0	0	56 (100)	57	38 (40)	0	1 (1)	57 (59)	96
Normandie	0	0	0	31 (100)	31	8 (33)	0	0	16 (67)	24
Nouvelle-Aquitaine	1 (2)	2 (4)	10 (21)	35 (73)	48	11 (20)	4 (7)	16 (30)	23 (43)	54
Occitanie	1 (2)	0	0	50 (98)	51	16 (30)	0	0	37 (70)	53
Pays de la Loire	0	3 (9)	5 (14)	27 (77)	35	10 (33)	3 (10)	9 (30)	8 (27)	30
Provence-Alpes-Côte d’Azur	2 (5)	0	0	36 (95)	38	17 (50)	0	0	17 (50)	34
Total	10 (2)	11 (2)	33 (7)	446 (89)	500	165 (33)	15 (3)	53 (11)	267 (53)	500
National compliance	10 (100)	4 (36)	0	16 (4)		155 (94)	3 (20)	4 (7.5)	17 (6)

## Discussion

This study describes the main lifestyle factors related to endoparasites in cats and dogs and the associated deworming behaviors of their owners across the various regions of Metropolitan France (European France). The results of this survey indicate that, among the regions, the majority of dogs and cats share lifestyle factors associated with a high risk of endoparasite transmission and infection according to the ESCCAP-based risk categories. However, the recommended deworming frequency (monthly) for those dogs and cats was largely not followed (only 4% for dogs and 6% for cats). Selection bias may have affected the classification of the animals. This survey was designed to randomly select the study population, but owners particularly interested in the subject may have been more likely to complete the survey. Likewise, other factors not addressed by the questionnaire may have substantially affected the deworming behavior of the owner or the deworming advice of veterinarians and thereby affecting our appraisal of compliance.

Several determinants are involved in the process of endoparasite infection and transmission. As some endoparasites are transmitted to dogs and cats by vectors or by direct or indirect contact with intermediate hosts, the distribution of the parasites may be limited to landscapes in which host and vectors share the same habitat, resulting in large differences in the risk of infection from region to region. Moreover, the parasite burden among different individuals is determined by multiple factors related to their lifestyle, as highlighted in previous studies [15, 16, 44–46]. Nevertheless, the lifestyle of pets may substantially change over time and within countries. In France, the risk factors associated with endoparasites in dogs and cats have been scarcely studied [20, 21, 47]. A continued surveillance program of pets for endoparasite prevalence, lifestyle and deworming rates may benefit public health and welfare of pets.

From our findings, among the most frequent lifestyle factors associated with low deworming compliance were: (i) the contact with other dogs, slugs, snails and preys; and (ii) the interaction with children and elderly. Nevertheless, these factors were approached through broad questions that may contribute to placing a large part of the dog population at category D. Therefore, further studies focused on the factors associated with the risk of pets endoparasites should enhance the precision of the lifestyle questionnaires to avoid potential selection bias when grouping the animals according to recognized risks. Nevertheless, although no specifics were obtained on the ages and the immunological status of the population surveyed in this study, the important zoonotic risk posed by *Toxocara* spp. on children and immune suppressed individuals is a concern that should guide the implementation of a comprehensive deworming protocol for pets in close contact with these populations.

This study documented low deworming levels which may be related to suboptimal client education and deworming protocols among the veterinary community, as reported by previous studies [48–51]. From our findings, dog owners reportedly dewormed their animals only 2.28 times a year on average. In all the regions the deworming frequencies were well below the frequencies advised. The highest deworming frequency was 3 times per year recorded in the Bourgogne-Franche-Comté region. However, because of the presence of *E. multilocularis*, pet owners should be more concerned about deworming in this area. The same tendency was observed for cats, largely categorized in category D (53%). Cat owners dewormed on average only 2.25 times per year instead of the monthly deworming recommendation for cats in this risk group. However, cats are considered poor hosts of *E. multilocularis.* Excreting only a few eggs in the environment, the risk of transmission is considerably reduced [52].

As pointed out above, although AE is a low incidence disease, the number of cases increased in Europe and specifically in France over the past few decades [25, 26]. Therefore, a special monitoring program for *E. multilocularis* has been developed in France through the National Reference Laboratory (NRL) for *Echinococcus* spp. in Nancy and the National Reference Centre (NRC) in Besançon. Although there is no legal reporting obligation of AE cases, the NRC has developed a network for recording AE cases between hospital centers, hospital pharmacies, and laboratories of pathology and parasitology throughout France [25, 26]. Living in rural areas is a factor frequently associated with AE cases. The majority of the population surveyed in this study reported living in rural areas and towns and owning pets that have contact with children and elderly people. The deworming frequencies recorded, however, were much lower than the advised frequencies. These findings highlight the importance of developing and implementing deworming guidelines adaptable to different pet’s lifestyle scenarios.

Otherwise, regarding the spread of *E. granulosus*, although a recent study indicated that the already low incidence of CE decreased between 2005 and 2014 [53], the estimated incidences in Corse and Provence-Alpes-Côte d’Azur regions remain the highest in France. Moreover, the parasite still affects intermediate hosts at low prevalence, mainly sheep and pig herds from the south-eastern and Corse regions, respectively. [30]. Therefore, the infection risk for dogs is still present and should be a primary concern for deworming strategies.

The southern and Corse regions, and French administered territories as well, have been identified as endemic areas for *D. immitis* [19, 33, 34]. Nevertheless, despite the risk encountered by dogs and cats of those regions, owners seem largely unconcerned as revealed by the deworming rates recorded in southern regions. However, the implementation of other means of *D. immitis* prevention, such as repellents, may hide an alternative preventive behavior of owners. Regarding *A. vasorum,* known as the “French heartworm”, which is mainly present in northern, southern and central regions (Ile de France), this parasite is considered to have national distribution [18, 31, 32]. Therefore, across the country, dog owners and veterinarians should be aware of the important risk that *A. vasorum* represent for dogs in contact with intermediate hosts such as slugs and snails. Regarding the risk of thelaziosis, during the last decade the parasite has been identified in new areas within France. The expansive spread of the parasite across the country could be explained as a consequence of the large dispersal of wild host reservoirs [54] and the suitability of the vector *P. variegata* thriving all over the country [55].

Finally, while there is little evidence supporting endoparasite resistance to anthelmintic drugs of dogs and cats, and multiple classes of anthelmintics are available for many species of nematodes, there is an increasing concern for minimizing this potential threat [56]. Therefore, the correct frequency and usage of antihelminthics is a priority for the development of effective and sustainable control strategies.

The low occurrence of deworming recorded during this survey highlight the importance of conducting future studies to investigate the changing deworming behaviours of owners and the deworming advice of veterinarians, especially for high-risk populations and recognized endemic regions. Increasing the owner’s and veterinarian’s compliance with deworming recommendations may significantly enhance the health and welfare of companion animals thereby reducing zoonotic risks [57, 58].

## Conclusions

The results of this survey highlight the low deworming rates of dogs and cats within the French metropolitan territory. Independent of the lifestyle of the dogs and cats surveyed, the deworming behavior of owners did not match the advised guidelines needed to reduce the potential risk of endoparasites infection and transmission, and did not adequately increase in frequency as risk increased. Future studies are warranted to develop, promote and evaluate effective and regular deworming strategies based on the lifestyle of the pets. In the meantime, veterinarians and pet owners should implement the risk assessment and deworming guidelines provided by ESCCAP.

## Supplementary information

**Additional file 1: Text S1.** French translation of the abstract.

## Data Availability

The datasets supporting the conclusions of this article are included within the article. Due to commercial confidentiality of the research, data not included in the manuscript can only be made available to bona fide researchers subject to a non-disclosure agreement.

## Abbreviations

ESCCAP: European Scientific Counsel Companion Animal Parasites
AE: Alveolar Echinococcosis
CE: Cystic Echinococcosis
NRL: National Reference Laboratory
NRC: National Reference Centre
