# Supplementary material for: French national survey of dog and cat owners on the deworming behaviour and lifestyle of pets associated with the risk of endoparasites
Source: Parasit Vectors. 2019 Oct 14;12:480. doi: 10.1186/s13071-019-3712-4 (PMC6792328; doi:10.1186/s13071-019-3712-4)
Supplement: Supplementary file 1 — Additional file 1: Text S1. French translation of the abstract. [file 13071_2019_3712_MOESM1_ESM.docx]

**Enquête nationale française réalisée auprès de propriétaires de chiens et de chats sur les habitudes de vermifugation et sur le mode de vie des animaux de compagnie associés aux risques d’endoparasitisme**

Clarisse Roussel^1^*, Jason Drake^2^, Juan Manuel Ariza^3^

^1^Elanco Santé Animale, 24 boulevard Vital Bouhot, CS 50004, 92521 Neuilly sur Seine Cedex, France

^2^Elanco Santé Animale, 2500 Innovation Way, Greenfield, IN 46140, Etats-Unis

^3^Chercheur indépendant

*Correspondance: [ariza_clarisse@elanco.com](mailto:ariza_clarisse@elanco.com)

Adresses électroniques:

Clarisse Roussel: [ariza_clarisse@elanco.com](mailto:ariza_clarisse@elanco.com)

Jason Drake: [drake_jon_j@elanco.com](mailto:drake_jon_j@elanco.com);

Juan Manuel Ariza: [juanmanuelariza@gmail.com](mailto:juanmanuelariza@gmail.com)

**Résumé**

**Contexte:** L’endoparasitisme des chiens et des chats est un sujet de préoccupation majeure en santé animale et publique. En France, peu d’informations relatives aux habitudes de vermifugation ou à la fréquence d’apparition des facteurs de risque sont connues, même si le mode de vie de l’animal ou encore la répartition géographique des parasites jouent un rôle important sur leur transmission. L’European Scientific Counsel Companion Animal Parasites (ESCCAP) recommande une vermifugation adaptée au mode de vie de l’animal prenant en compte le risque d’infestation individuel et zoonotique. Les objectifs de cette étude étaient d’explorer les facteurs de risque liés à l’endoparasitisme des chiens et chats français et d’évaluer la conformité des fréquences de vermifugation.

**Méthodes:** Les données ont été extraites d’une base de données d’une récente enquête européenne. Les animaux étaient classés en différentes catégories de risque identifiées par l’ESCCAP. Les fréquences de vermifugation reportées dans les différentes régions françaises ont été explorées et confrontées aux fréquences de vermifugation recommandées.

**Résultats:** La majorité des chiens et des chats avaient plus de 6 mois, avaient accès à l’extérieur, avaient des contacts avec des enfants ou des personnes âgées et vivaient en zone rurale ou péri urbaine. La plupart des chiens étaient au contact d’autres chiens, d’escargots ou de proies (83%), mangeaient des escargots, des limaces, de l’herbe ou creusaient des trous dans le jardin (68%). La plupart des chats avaient une activité de chasse (57%), et attrapaient des proies (52%). 89% des chiens et 53% des chats appartenait à la catégorie D (haut risque). Cependant, les recommandations canines étaient très peu respectées (6%) et ce, quelle que soit la région étudiée. Concernant les chats de la catégorie A (faible risque), les recommandations étaient fortement respectées (94%), pour ceux de la catégorie D (haut risque), très peu respectées (6%).

**Conclusions:** Le respect des recommandations en terme de vermifugation est nécessaire afin de préserver la santé animale et de réduire les risques zoonotiques. D’autres études sont à réaliser pour investiguer de manière approfondie le respect des recommandations, l’efficacité des protocoles de vermifugation ainsi que les facteurs de risque associés à l’endoparastisme en France.
